# Enhanced ethanol production from brewer's spent grain by a *Fusarium oxysporum *consolidated system

**DOI:** 10.1186/1754-6834-2-4

**Published:** 2009-02-10

**Authors:** Charilaos Xiros, Paul Christakopoulos

**Affiliations:** 1 BIOtechMASS Unit, Biotechnology Laboratory, Chemical Engineering Department, National Technical University of Athens, Iroon Polytechniou St, Zografou Campus, 15700, Athens, Greece

## Abstract

**Background:**

Brewer's spent grain (BG), a by-product of the brewing process, is attracting increasing scientific interest as a low-cost feedstock for many biotechnological applications. BG in the present study is evaluated as a substrate for lignocellulolytic enzyme production and for the production of ethanol by the mesophilic fungus *Fusarium oxysporum *under submerged conditions, implementing a consolidated bioconversion process. Fermentation experiments were performed with sugar mixtures simulating the carbohydrate content of BG in order to determine the utilization pattern that could be expected during the fermentation of the cellulose and hemicellulose hydrolysate of BG. The sugar mixture fermentation study focused on the effect of the initial total sugar concentration and on the effect of the aeration rate on fermenting performance of *F. oxysporum*. The alkali pretreatment of BG and different aeration levels during the ethanol production stage were studied for the optimization of the ethanol production by *F. oxysporum*.

**Results:**

Enzyme yields as high as 550, 22.5, 6.5, 3225, 0.3, 1.25 and 3 U per g of carbon source of endoglucanase, cellobiohydrolase, β-D-glucosidase, xylanase, feruloyl esterase, β-D-xylosidase and α-L-arabinofuranosidase respectively, were obtained during the growth stage under optimized submerged conditions. An ethanol yield of 109 g ethanol per kg of dry BG was obtained with alkali-pretreated BG under microaerobic conditions (0.01 vvm), corresponding to 60% of the theoretical yield based on total glucose and xylose content of BG.

**Conclusion:**

The enzymatic profile of the extracellular extract from *F. oxysporum *submerged cultures using BG and corn cob as the carbon source was proved efficient for a successful hydrolysis of BG. The fermentation study carried out using sugar mixtures simulating BG's carbohydrates content and consecutively alkali-pretreated and untreated BG, indicates that BG hydrolysis is the bottleneck of the bioconversion process. However, a considerable bioconversion yield was achieved (60% of the theoretical) making this bioprocess worthy of further investigation for a potential commercial application.

## Background

To date, alcohol fuels have been produced on industrial scales by fermentation of sugars derived from wheat, corn, sugar beets, sugar cane and molasses. Lately the use of starch and sugar crops as raw materials in the biofuels industry has been for the subject of discussion. More and more scientific efforts are being made towards an efficient technology for the biological conversion of lignocellulosic materials to second-generation biofuels. The enzymatic hydrolysis of cellulosic materials to produce fermentable sugars has an enormous potential in meeting global bioenergy demand through the biorefinery concept, since agri-food processes generate millions of tonnes of waste each year, such as spent grain from brewing (BG) and corn cob (CC). Thus, several companies around the world are currently working toward developing technologies for producing cellulosic ethanol on a commercial scale.

BG is a by-product of the brewing process, consisting of the solid residue remaining after mashing and lautering. It consists primarily of grain husks and other residual compounds not converted to fermentable sugars by the mashing process. BG is the most abundant brewing by-product, corresponding to around 85% of total by-products generated [1] and is mainly used as low-value cattle food. The chemical composition of BG varies according to barley variety, harvest time, malting and mashing conditions, and the quality and type of adjuncts added in the brewing process [2,3].

The BG used in this study, as described previously [4], contains mainly hemicellulose in the form of arabinoxylans from the barley grain and cellulose. BG has the potential to serve as a low-cost feedstock for the production of ethanol since hemicellulose and cellulose content corresponds to 52% w/w of dry BG. Other substances such as proteins, lignin and fat are also present in BG in significant quantities.

Current technology for conversion of lignocellulose to ethanol requires chemical or enzymatic conversion of the substrate to fermentable sugars followed by fermentation by a microorganism. The large amounts of enzymes required for enzymatic conversion of hemicelluloses and cellulose to fermentable sugars impacts severely on the cost effectiveness of this technology. The physical support and the energy required for a fungus to grow and produce the desired metabolite is primarily provided by a substrate [5]. Generally, the production of cellulases and hemicellulases has been shown to be inducible and is affected by the nature of the substrate used in fermentation [6]. Therefore, the choice of an appropriate inducing co-substrate is of importance. CC, the central wooden core of maize (*Zea mays *ssp. mays L.), has been used as an excellent carbon source for enzyme production by fungi [7-9] and especially *Fusarium oxysporum *[10]. Nowadays, CC, a chip agricultural by-product, is an important source of the furfural, an aromatic aldehyde used in a wide variety of industrial processes.

Considerable research efforts have been made to improve conversion yields of lignocellulosic materials by the insertion of a pre-treatment step prior to the enzymatic hydrolysis. Dilute NaOH is an effective pre-treatment for lignocellulosic materials with relatively low lignin content of 10 to 18% [11]. The necessity and the suitability of this initial step in the bioconversion process has been previously analyzed and evaluated [4].

One-step conversion of lignocellulose degradation to ethanol with an organism capable of lignocellulose degradation and efficient fermentation (consolidated bioprocessing-CBP) would greatly enhance the cost effectiveness of bioethanol production [12]. *F. oxysporum *acquires the exceptional ability of bioconverting cellulose and hemicellulose directly to ethanol through the consecutive steps of hydrolysis of the polysaccharides and fermentation of the resulting oligosaccharides [13].

The ethanol production by the mesophilic fungus *F. oxysporum *by coupling solid state and submerged bioreactor fermentation was previously investigated. The simultaneous production of cellulolytic and xylanolytic enzymes in solid-state culture, the increase of BG's saccharification and the consequent enhancement of ethanol production upon alkali pretreatment of BG was also investigated [10]. The submerged culture technique is widely used for biotechnological applications as it is intrinsically less problematic (heat and oxygen mass transfer are much better, and culture homogeneity is usually superior) making it more reliable and reproducible, easier to monitor and to control key operational parameters, and it is more flexible [14].

The study of the fermentative performance of *F. oxysporum *is essential for the optimization of the bioconversion yield of the whole process. The fermentation of sugar mixtures simulating the composition of BG is a useful tool in order to observe the fermentative performance of the microorganism and predict its behaviour in the BG's hydrolysate [15]. In this study consolidated bioprocessing was implemented, taking advantage of the above-mentioned exceptional abilities of *F. oxysporum *with regard to the bioconversion of BG to ethanol. Both growth and production stages were carried out under submerged cultivation conditions. The effect of BG mixed with CC as the carbon source on the simultaneous production of cellulolytic and xylanolytic enzymes in submerged culture was investigated. Sugar mixtures simulating the composition of BG were used for the study of the fermentative performance of *F. oxysporum*. The fermentation of BG was also studied. The (previously investigated) increase of BG saccharification upon alkali pre-treatment was evaluated with regard to the enhancement of ethanol production under submerged conditions. The effect of the aeration level and the effect of the initial sugar concentration on ethanol production were also investigated.

## Results and discussion

### Evaluation of BG as a substrate for enzyme production by F. oxysporum under submerged cultivation

The production of an efficient lignocellulolytic enzyme system by *F. oxysporum *is essential for the bioconversion of BG. The optimization of enzyme production focused on the incorporation of BG as the carbon growth substrate. Other factors (culture pH, mineral medium, culture temperature) affecting the lignocellulolytic production by *F. oxysporum *during the growth stage under submerged conditions have been previously optimized [16]. Table 1 summarizes the activities for the major degrading lignocellulolytic enzymes (xylanase and endoglucanase) obtained after five days of aerobic growth when *F. oxysporum *was grown using different BG concentrations. As is shown, enzymatic activities per volume of culture increase when the initial BG concentration increases, while the specific activities (Units per dry material) decrease. The initial BG concentration 4% w/v was chosen as the optimum with regard to a successful exploitation of the material for sufficient enzymatic production. High cellulolytic and hemicellulolytic activities are necessary for a successful bioconversion process.

**Table 1:** Effect of initial BG concentration on extracellular enzyme activities

BG concentration	Xylanase		Endoglucanase	
	U mL^-1^	U g^-1^	U mL^-1^	U g^-1^

1%	14 ± 9	1400 ± 459	7 ± 0.6	700 ± 59

2%	36 ± 5	1800 ± 266	10 ± 0.7	500 ± 34

3%	52 ± 3	1733 ± 109	11 ± 1	367 ± 40

4%	58 ± 1	1450 ± 31	12 ± 2	300 ± 44

5%	62 ± 4	1240 ± 79	12 ± 2	220 ± 36

6%	66 ± 1	1100 ± 9	14 ± 0.4	233 ± 6

The enzyme activities are obtained after 120 h of growth. All experiments were carried out in Erlenmeyer flasks, in triplicate. Assays were carried out in duplicate.

**Figure 1:**
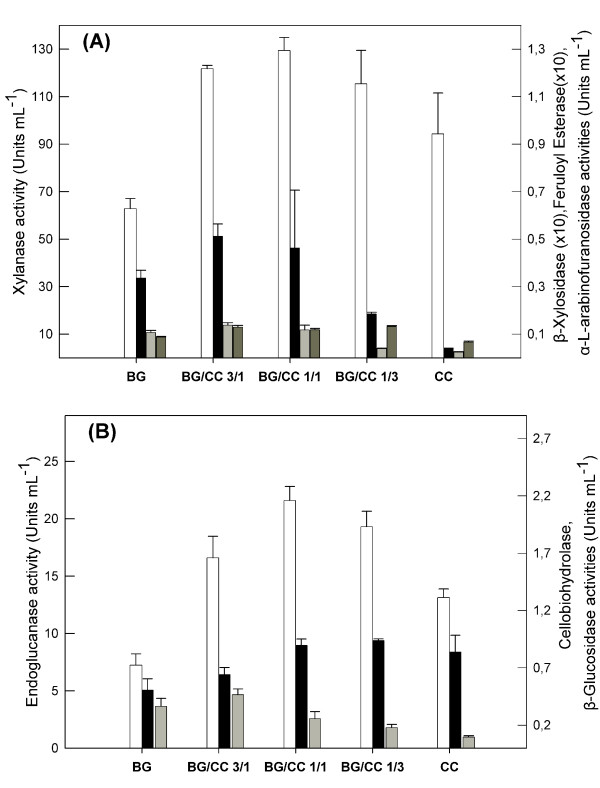
**Effect of the carbon source on the production of lignocellulolytic enzymatic activities by *F. oxysporum***. (A) hemicellulolytic (white – xylanase, dark grey – α-L-arabinofuranosidase, black – xylosidase, light grey – feruloyl esterase) and (B) cellulolytic enzymes (white – endoglucanase, dark grey – β-glucosidase, black – cellobiohydrolase) activities produced by *F. oxysporum *grown under submerged conditions for 5 days. The initial culture pH was 6.0 and the temperature was set at 30°C. Values are the mean of three determinations and vertical error bars represent standard deviation.

The use of BG as a raw material in the bioethanol production stage requires higher enzyme yields than those obtained using BG as a single substrate. CCs have been previously evaluated as a very good carbon source for the production of hemicellulolytic enzyme activities [16]. Thus CCs were chosen as an additional carbon source for the enhancement of enzymatic production. The influence of individual CC and BG as well as their combination in various ratios on the hemicellulolytic (Figure 1A) and cellulolytic (Figure 1B) enzyme production by *F. oxysporum *is shown in Figure 1. The initial concentration of the carbon source in the culture medium was 40 g L^-1^. The xylanase production was doubled and the production of endoglucanase was enhanced up to three times when one part of CC and one part of BG was used as carbon source, compared with production when only BG was used. Analogous results were obtained for feruloyl esterase, cellobiohydrolase and β-D-xylosidase, reaching 50% higher activities. Production of auxiliary enzymes such as α-L-arabinofuranosidase and β-glucosidase was not affected at a measurable level.

The CC as a co-substrate enhanced enzyme production as all the investigated enzymes showed maximum activities when a mixture of BG and CC was used. The major degrading enzymes, endoxylanase and endoglucanase, reached maximum activities after 120 h of cultivation. At this point and for BG/CC ratio 1/1, activities as high as 129, 22, 0.26, 0.12, 0.9, 0.012 and 0.05 U mL^-1 ^carbon source for xylanase, endoglucanase, β-D-glucosidase, α-L-arabinofuranosidase, cellobiohydrolase, feruloyl esterase and β-D-xylosidase were obtained, respectively. Xylanase activity was measured using birchwood and oat spelt xylan as the substrate in order to facilitate comparison with previous studies. The assay using oat spelt xylan showed a 30% higher activity under the same assay conditions. In Table 2 a comparison is made concerning enzyme production between *F. oxysporum *and other microorganisms grown on BG or CC in submerged cultivation [17,18]. The xylanase activity obtained here is on same levels as that reported by Christakopoulos and colleagues [16] for a CC concentration 3.5% w/v. In that study, xylanase activity decreases as the concentration of CC increases above 2% w/v. Thus, only data up to 3.5% w/v are reported. The lignocellulolytic activities obtained in the present study compare favourably with all other previous studies and confirm that *F. oxysporum's *enzymatic system has great potential for the hydrolysis of lignocellulosic substrates. These results also show that BG is a promising substrate for industrial and commercial-scale bioconversion processes under submerged conditions.

**Table 2:** Enzyme production under submerged conditions using corn cobs or/and brewers spent grain as the carbon source.

Xylanase		Endoglucanase		FAE				
U mL^-1^	U g^-1^	U mL^-1^	U g^-1^	U mL^-1^	U g^-1^	Microorganism	Carbon source	Reference
129/168*	3225/4200*	22	550	0.12	3	*F. oxysporum *F3	BG/CC	Present study

0.67	67			0.08	8	*S. avemitilis*	BG	[17]

16	1600	0.15	15			*Streptomyces sp. AMT-3*	BG	[18]
9.1	910	0.24	24				CC	

170*	4857*	-	-	-	-	*F. oxysporum *F3	CC	[16]

(*) Indicates that the assay was carried out with oat spelt xylan. For the present study the activities presented here are obtained under optimum conditions. All experiments were carried out in Erlenmeyer flasks, in triplicate. Assays were carried out in duplicate.

### Ethanol production in submerged bioreactors from sugar mixtures simulating the carbohydrate content of BG

The sugar content of BG has been previously determined [4]. Cellulose and hemicellulose are the main polysaccharides in the material and correspond to 52% w/w of dry BG. Xylose, arabinose and glucose in a ratio of 2:1:1 represent approximately 97% of the total reducing sugars in BG polysaccharides. The sugar mixtures simulating the composition of the BG were prepared and studied in order to determine the utilization pattern that could be expected during the fermentation of the cellulose and hemicellulose hydrolysate of BG. Thus the effect of different conditions on the initial fermentation rate, on the assimilation rate of each sugar and on ethanol yield was determined, leading to a deeper understanding and better design of the bioconversion process of BG. The sugar mixture fermentation study focused on the effect of initial total sugar concentration, and the effect of aeration rate on the fermenting performance of *F. oxysporum*.

### Effect of aeration level on ethanol production

**Figure 2:**
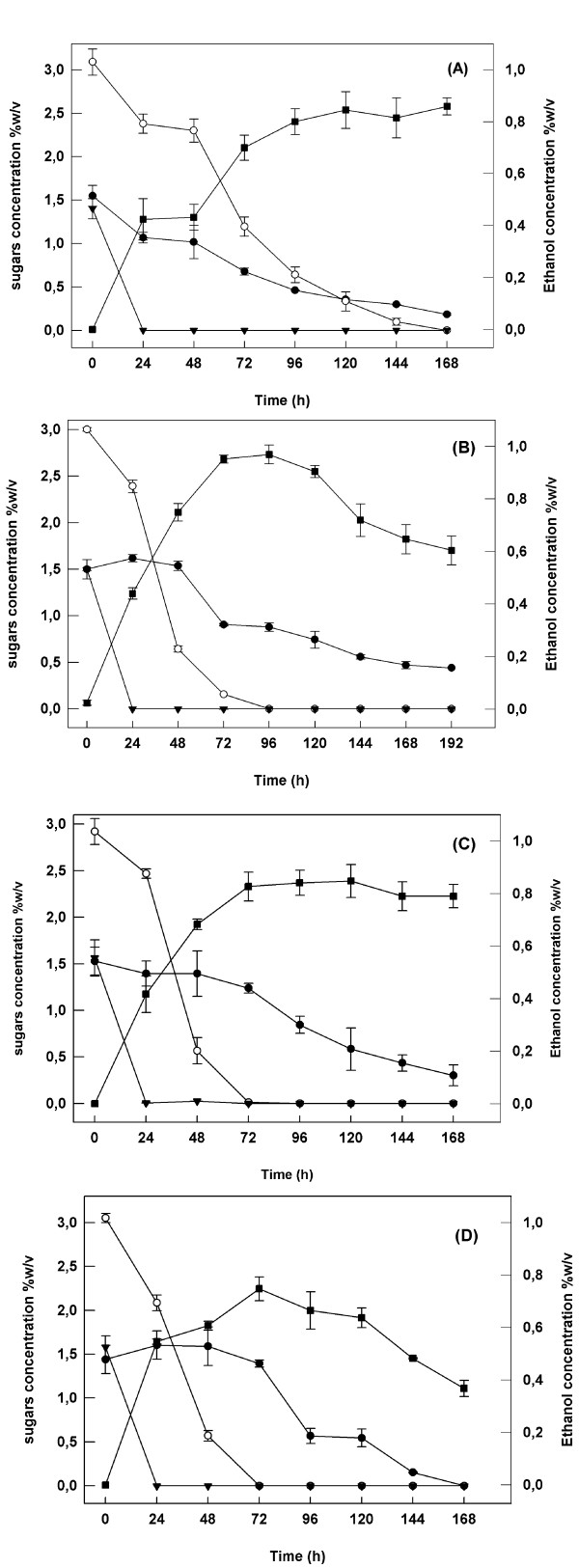
**Time course profile of ethanol production and sugars assimilation in various aeration rates by *F. oxysporum***. A) 0 vvm, B) 0.1 vvm, C) 0.2 vvm, D) 0.4 vvm. The concentration of the sugar mixture and the ratio of the sugars (glucose/xylose/arabinose) in the mixture was 6% w/v and 1/2/1 respectively in all cases (black circle – Arabinose, open circle – xylose, black triangle – glucose, black square – ethanol). Experiments were performed under submerged conditions in cylindrical bioreactors of 1 L working volume. The initial culture pH was 6.0 and the temperature was set at 30°C. Values are the mean of three determinations and vertical error bars represent standard deviation.

In Figure 2, a time course of ethanol production in different aeration rates (0 vvm, 0.1 vvm, 0.2 vvm, 0.4 vvm) and of the sugars' consumption during the fermentation is presented. The initial sugar mixture concentration was 6% in all cases. The ethanol production rate during the first 24 h is similar in all cases except the case of 0.4 vvm, where the initial production rate is higher but the ethanol yield remains at low levels. After the first 24 h we may assume that ethanol production rate follows the xylose assimilation rate. In all microaerobic conditions (0.1, 0.2, 0.4 vvm, Figure 2B, 2C, 2D, respectively) maximum ethanol production was observed after 72 h of fermentation. On the contrary, under anaerobic conditions, ethanol production was much slower, reaching highest levels after 120 h of fermentation. The maximum ethanol production that was observed after 96 h of fermentation, under an air flow rate of 0.1 vvm, reached a concentration of 10 g L^-1^, corresponding to a yield equal to 44% of the theoretical, based on total initial concentration of fermentable sugars (glucose and xylose) in the fermentation broth.

In all cases glucose was consumed during the first 24 h, while arabinose consumption was very low and did not affect ethanol production. This is in accordance with earlier studies that report that L-arabinose is not significantly convertible to ethanol by *F. oxysporum *[19] and by most of yeasts [20]. Xylose was assimilated and not detected in the culture media after 72 h of fermentation when the culture was provided with small amount of air (0.1 and 0.2 vvm). The slower assimilation of xylose under anaerobic conditions is in agreement with earlier studies, which show that under anaerobic conditions *F. oxysporum *utilizes only glucose and a very small fraction of the xylose when it is cultivated on a glucose-xylose mixture. A small aeration is needed because of redox imbalance in fungal metabolism of xylose to ethanol. [21]. Other studies also confirm that when yeasts grow on glucose-xylose mixtures, xylose is only converted if the glucose concentration is sufficiently low not to inhibit xylose conversion [22], probably because glucose may inhibit the transport of xylose into the cell [23].

### Effect of initial concentration of sugars mixture on ethanol production

In preliminary experiments carried out in Erlenmeyer flasks, sugar concentrations from 2% up to 8% were tested (data not shown). Ethanol production increased as the sugar concentration increased up to 6%. For concentrations above 6% w/v no statistically significant increase of ethanol production was observed. Furthermore, the ethanol yield (g EtOH per g of sugar added) decreased as the sugar concentration increased above 6% w/v. Thus initial sugar concentrations up to 6% w/v were used in fermentation experiments performed in bioreactors.

In order to investigate the effect of initial sugar concentration on the initial fermentation rate, on the assimilation rate of each sugar and on ethanol yield, initial sugar concentrations from 3% w/v up to 6% w/v were used. The glucose/xylose/arabinose ratio was kept stable and equal to 1:2:1, simulating as in all other experiments BG's hydrolysate. All fermentations were carried out in 2 L bioreactors under oxygen-limited conditions (0.1 vvm).

**Figure 3:**
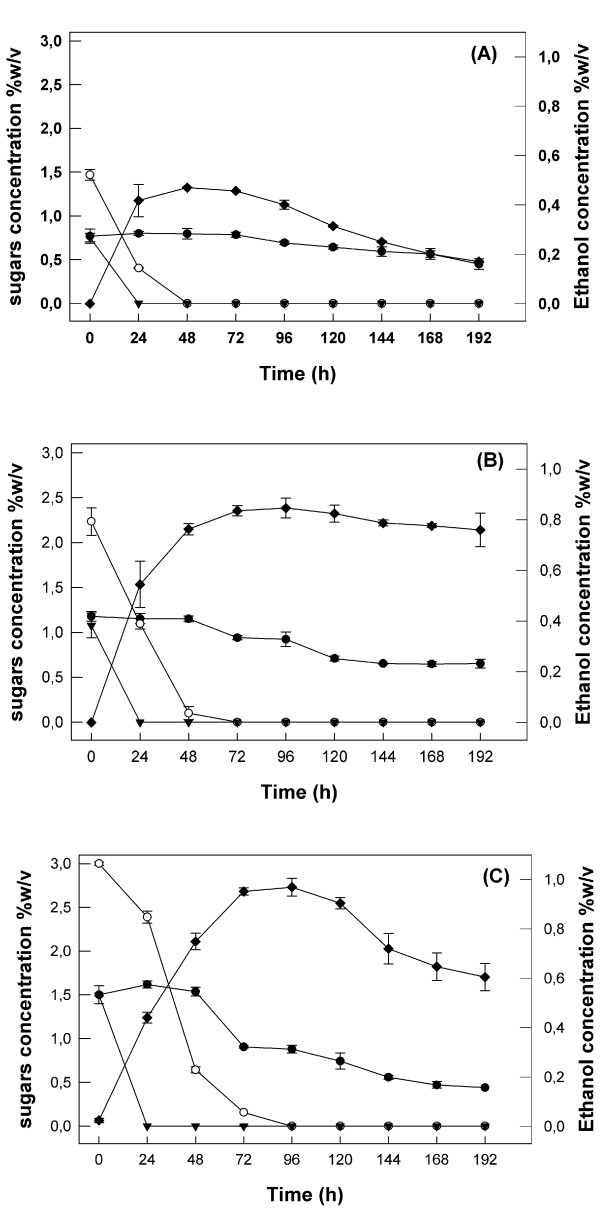
**Time course profile of ethanol production and sugars assimilation in various sugar mixtures concentrations**. A) 3% w/v B) 4.5% w/v C) 6% w/v. The ratio of the sugars of the mixture was glucose/xylose/arabinose 1/2/1 in all cases (black circle – Arabinose, open circle – xylose, black triangle – glucose, black square – ethanol). Experiments were performed under submerged conditions in cylindrical bioreactors of 1 L working volume using microaerobic conditions (aeration level 0.1 vvm). The initial culture pH was 6.0 and the temperature was set at 30°C. Values are the mean of three determinations and vertical error bars represent standard deviation.

*F. oxysporum*, as shown in Figure 3 where a time course of ethanol production and as well the sugar consumption during fermentation are presented, achieved almost maximum ethanol production after 72 h in experiments with initial concentrations 4.5% w/v and 6% w/v (0.84% w/v and 0.95% w/v respectively), while for a sugar concentration of 3% w/v, maximum production (0.48% w/v) achieved after 48 h.

The ethanol production rates for the first 24 h are on the same levels as those observed in experiments described above, except for the concentration 4.5% w/v where a slightly higher rate is obtained. The best ethanol yield (48% of the theoretical) was achieved using an initial sugar mixture concentration of 4.5%. In all cases glucose is assimilated within 24 h of fermentation. Glucose has been possibly consumed even earlier, especially in the cases of initial concentrations of 3% and 4.5%, taking into account the glucose uptake rates reported in earlier studies for *F. oxysporum *[21,24]. As shown in Figure 3C, (1.5% w/v glucose for total sugar concentration 6% w/v), glucose presence in the fermentation broth probably influences xylose consumption, causing a delay in its utilization in comparison with the two other cases (Figure 3A and 3B). This delay has been also observed when the aeration rate was 0.2 vvm (Figure 2C). Xylose conversion to ethanol starts at lower glucose concentrations, indicating that the xylose uptake rate may be affected by the high initial glucose concentration [23].

### Ethanol production form BG, implementing a consolidated bioprocess under submerged conditions

The study of BG's fermentation by *F. oxysporum *was carried out using initial BG concentration 7.5% w/v with regard to the optimum initial sugar mixture concentration (4.5% w/v). The fermentable sugar (glucose and xylose) content of 7.5 g BG is 3.9 g as determined earlier [4]. Alkali pretreated material was used for the enhancement of ethanol production, since it has been previously shown that enzymatic hydrolysis of alkali-pretreated BG using enzymes extracted from *F. oxysporum *grown under solid-state conditions was increased by about 50% compared with non-pretreated material [10].

**Figure 4:**
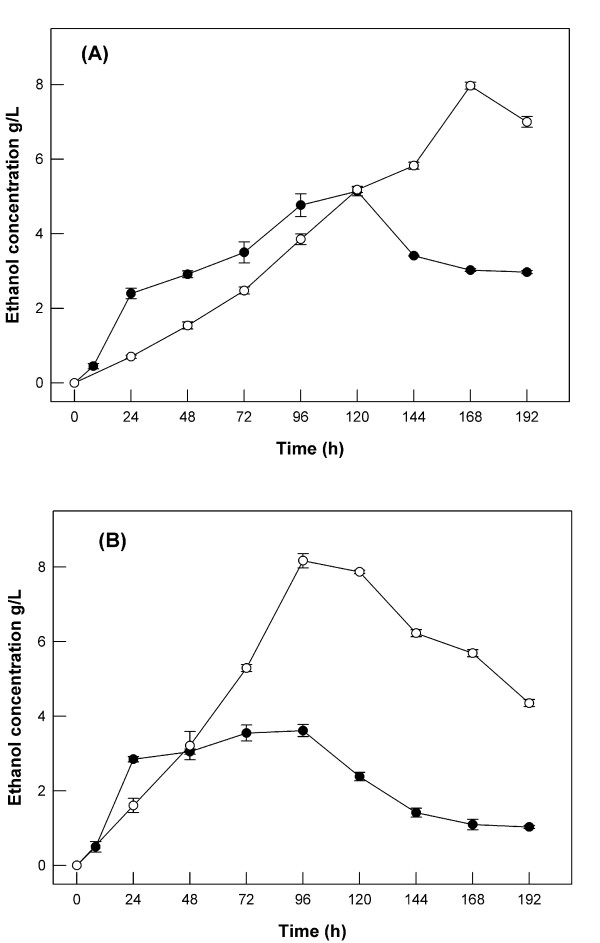
**Ethanol production by *F. oxysporum***. Under anaerobic conditions (A) and micro-aerobic conditions (B). BG concentration was 75 g L^-1 ^in all cases. All fermentations took place in bioreactors of 1 L working volume at 30°C and pH 6.0. Values are the mean of three determinations and vertical error bars represent standard deviation. The NaOH concentration for pretreatment was 10 g NaOH per 100 g dry BG (black circle – Non pretreated spent grain, open circle – pretreated spent grain).

Ethanol production from alkali-treated and untreated BG by *F. oxysporum *in 2 L bioreactors under anaerobic and microaerobic conditions is presented in Figure 4A and 4B respectively. When alkali-pretreated BG was used the ethanol production increased about two times under anaerobic conditions, while under microaerobic conditions (air flow 0.1 vvm), ethanol production increased about 2.5 times. The maximum ethanol yield under anaerobic conditions was achieved after 168 h. This very low production rate indicates that the ethanol increase was probably caused by the medium concentration. The ethanol production rate under microaerobic conditions was almost double compared with anaerobic conditions, a fact in accordance with the results obtained from the simulation experiments. The ethanol yield under microaerobic conditions reached 110 g ethanol Kg^-1 ^dry BG, corresponding to 60% of the theoretical yield based on the total glucose and xylose composition of BG.

In Table 3, a comparison of initial ethanol production rates between simulation experiments and BG fermentation is presented. As is shown the initial rate for BG fermentation is considerably lower. Taking into account the fact that no glucose accumulation was observed during fermentation and the released xylose was more slowly consumed and was detected only in traces after the fifth day of fermentation, we may conclude that BG hydrolysis is the bottleneck of the consolidated bioconversion process. The seemingly higher initial rate for the conversion of untreated material compared with pretreated material could be explained by the conversion to ethanol of sugars not belonging to polymers (cellulose, arabinoxylan) of BG. These sugars (starch, glucans), present in most lignocellulosic substrates, are not available in the pretreated material possibly because they are degraded by NaOH during the pretreatment.

**Table 3:** Initial ethanol production rates in sugar mixtures and BG fermentation experiments.

Fermentation substrate	Sugar mixture 4.5% w/v	Untreated BG 7.5% w/v	Pretreated BG 7.5% w/v	Untreated BG 7.5% w/v	Pretreated BG 7.5% w/v
Aeration rate	0.1 vvm			0 vvm	

Initial ethanol production rate (mg L^-1 ^h^-1^)	229 ± 37	121 ± 22	67 ± 7	100 ± 6	33 ± 1

The bioconversion yield achieved here is on the same levels as others reported for lignocellulosic substrates, although the high hemicellulose content (40% w/w per dry BG, mainly xylose and arabinose in a ratio 2:1) of the BG used in the present study demonstrates the difficulties for the bioconversion of this material. To the authors best knowledge this is the first study regarding bioconversion of BG to ethanol to implement a consolidated bioprocess under submerged conditions.

BG bioconversion was previously studied by coupling solid-state cultivation for the growth and enzyme production stage and submerged cultivation for the ethanol production stage by *Neurospora crassa *[4] and by *F. oxysporum *[10]. The ethanol yield obtained here is the best reported so far for the bioconversion of BG to ethanol. As is shown in Table 4, which summarizes the comparison between the two technological approaches, using *F. oxysporum *as the fermenting microorganism the ethanol yield obtained here is considerably higher. The difference between initial enzyme activity seems to be the main reason that leads to these results, except for the effect of fungal morphology and physiology in different cultivation conditions. Apart from the latter, which merits further investigation, this comparison is another clue about the effect of hydrolysis rate on the rate of bioconversion process, indicating that the lower enzymatic activities achieved in the fermentation stage from the solid-state culture, in comparison with those from the submerged culture, may result in a lower hydrolysis rate during the bioconversion process.

**Table 4:** Bioconversion of spent grain to ethanol using two different technical approaches.

Growth stage	Biomass (g L^-1^)	Enzyme activities (Units mL^-1^)		Initial Ethanol production rate (mg L^-1 ^h^-1^)	Ethanol yield (g EtOH kg^-1 ^dry BG)	Theoretical ethanol yield* g EtOH kg^-1 ^dry BG	Reference
						
		Endoglucanase	Xylanase				
Submerged culture	1.52	8.8	52	67	109	195	Present study

Solid state culture	2.55	0.6	9.8	29	65	195	[10]

*The theoretical ethanol yield is based on total glucose and xylose content of dry material

## Conclusion

In conclusion, it was possible to control simultaneous production of cellulolytic and hemicellulolytic enzymes from *F. oxysporum *and generate a multi-enzymatic system capable of hydrolyzing lignocellulosic substrates using a growth medium consisting of BG, CC and a mineral source under submerged conditions. The fermentation study using sugar mixtures simulating BG's carbohydrate content led to useful conclusions concerning the bioconversion of BG to ethanol. Hydrolysis seems to be the bottleneck of the process while the fermentative performance of *F. oxysporum *was satisfactory, achieving a bioethanol yield of 109 g kg^-1 ^of dry material, which corresponds to 60% of the theoretical yield. The high pentose content of BG and the ability of *F. oxysporum *to ferment xylose make this process worthy of further investigation with regard to the fungal morphology and physiology under the bioprocess conditions for a deeper understanding of the bioconversion process.

## Methods

### Carbon sources

CCs were supplied by the Agricultural University of Athens. The material was chopped in a laboratory hammer mill to a particle size smaller than 3 mm. BG was provided by Athens Brewery S.A. The material was frozen immediately after collection and was stored at -18°C. Before use it was dried at 65°C for 48 h and was chopped in a laboratory hammer mill to a particle size smaller than 5 mm.

### Microorganism

The fungus *F. oxysporum *F3 isolated from cumin [25] was used in the present investigation. The fungus was grown on potato-dextrose-agar (PDA) slants for 5 days at 30°C. The slants were maintained as a stock culture at 4°C.

### Inoculum

For inoculum preparation, 15 mL deionized sterile water, containing 100 μl Tween 80, was added to a PDA slant of the stock culture and aliquots (5 mL) of the mixture were used to inoculate 250 mL Erlenmeyer flasks (sterilized at 110°C for 35 min and cooled prior to inoculation) containing 100 mL of the following mineral medium: (in g L^-1^) 1.00 KH2PO4, 0.30 CaCl2·2H2O, 0.30 MgSO4·7H2O, 10.0 (NH4)2HPO4, 6.94 NaH2PO4 2H2O, 9.52 Na2HPO4 2H2O [26], supplemented with 20 g L^-1 ^CC and 20 g L^-1 ^BG. The pH was adjusted to 6.0. The flasks were incubated at 30°C for 3 days in an orbital shaker (250 rpm) for mycelium production.

### Enzyme production optimization experiments under submerged conditions

Submerged culture was carried out in 250 mL Erlenmeyer flasks containing 4 g of carbon sources (BG and CC in different ratios according to the optimization process of enzyme production) and 100 mL of mineral medium. Prior to sterilization, the initial pH of the medium was adjusted to 6.0. The medium was sterilized at 110°C for 40 min. The production culture medium was inoculated with 5 ml of 72 h-old culture (prepared as described above). The flasks were incubated at 30°C for 7 days. At different time intervals aliquots of 5 mL were aseptically withdrawn and used for enzyme estimation.

### Ethanol production in bioreactors

For the fermentation (ethanol production) stage, 2 L thermoregulated double-jacket cylindrical agitated bioreactors (B. Braun Biotech International, Germany) with 1 L working volume were used. The initial concentration of BG was in all cases 75 g L^-1^, while temperature and agitation speed was 30°C and 180 rpm, respectively. The sterilization of bioreactors was performed in an autoclave (Sanyo, Japan) at 121°C for 20 min. When pretreated spent grain was used, NaOH was added to the bioreactor prior to sterilization in a liquid-to-solid ratio 8:1 (w/w). The sterilization and pretreatment of the material was carried out in one step in the autoclave (Sanyo, Japan) at 121°C for 30 min. After pretreatment the slurry was neutralised *in situ *with the addition of drops of 1 M sulphuric acid and pH was adjusted to 6.0 before the addition of 400 mL of fungal culture grown aerobically for 96 h under submerged optimized conditions. The aeration rate and pH were kept stable during fermentation. The airflow to bioreactors was controlled and provided by a mass flow meter (Bronkhorst high-tech BV, The Netherlands). Sampling was carried out aseptically using a sterile incorporated sampler.

The experiments with sugar mixtures as the carbon source in the ethanol production stage were carried out with glucose, xylose and arabinose in 1:2:1 constant ratio, simulating the carbohydrates content of BG's hydrolysate. Various initial concentrations of total sugars (from 2% w/v up to 8% w/v) and different aeration levels (0, 0.1, 0.2, 0.4 vvm) were studied for the optimization of the process.

### Enzyme assays

At different time intervals aliquots of 5 mL were aseptically withdrawn and used for estimation of enzymes. The suspended materials and the fungal biomass were separated by centrifugation (10000 g for 10 min). The clarified supernatant was used as the source of the enzyme. One unit (U) of enzyme activity was defined as the amount of enzyme required to liberate 1 mol of product per min, at assay temperature. Yields were expressed as U per mL of culture or as U per g of dry carbon source for all enzymatic activities assayed. All enzyme assays were carried out as described previously [10]. Xylanase activity was measured using birchwood (1% w/v) and oat spelt xylan (1% w/v) as the substrate under the same assay conditions.

### Biomass estimation

The biomass content was measured by the colorimetric method of Scotti et al [27], based on glucosamine estimation of the fungal cell wall.

### BG analysis and pretreatment

BG analysis and pretreatment was carried out as described previously [10]. The pentose content was estimated after acid hydrolysis of BG with 7% (w/v) H2SO4 at 96°C for 3 h. The quantitative saccharification of BG was accomplished by acid hydrolysis with H2SO4 72% (w/w). All sugars contents were determined by HPLC as described below. The acid-insoluble lignin content was estimated after the sugars had been hydrolyzed and removed, by drying at 110°C and weighing the dry matter (ASTM D-1106).

### Ethanol, acetic acid and sugars determination

Ethanol and acetic acid determinations were made by column liquid chromatography (CLC). A Jasco (PU-987) HPLC system equipped with an ion-moderated partition chromatography column, Aminex HPX-87H (Bio-Rad), was used together with a Waters (410) refractive index detector and a UV detector. The flow rate of the mobile phase consisting of 5 mM H2SO4 was set to 0.6 mL min^-1 ^and the temperature to 45°C.

Glucose, arabinose, and xylose contents were determined by high pH anion exchange chromatography (HPAEC) using a Dionex electrochemical pulse amperometric detector (ED40) and separation was by isocratic elution on CarboPack PA1, 4 × 250 mm column (Dionex Corporation, USA) with a CarboPack PA1 guard column (Dionex Corporation, USA). The flow rate of the mobile phase consisting of 17.5 mM NaOH was set to 1 mL min^-1^. After each analysis the column was eluted with 200 mM NaOH. All samples were filtered (0.20 mm polyester syringe filter, Macherey-Nagel) prior to the analysis and diluted to reach concentrations from 20 to 100 M. Fucose (50 M) was used as an internal standard. All standards and dilutions were prepared with ultra-pure water (Millipore, France).

## Competing interests

The authors declare that they have no competing interests.

## Authors' contributions

CX carried out the experiments, participated in experimental design, analyzed the results and drafted the manuscript. PC conceived the study, designed and supervised the experiments, participated in results analysis and reviewed the manuscript. Both authors have read and approved the final manuscript.
